# Incidence and survival of childhood bone cancer in northern England and the West Midlands, 1981–2002

**DOI:** 10.1038/sj.bjc.6604837

**Published:** 2009-01-06

**Authors:** R Eyre, R G Feltbower, E Mubwandarikwa, H C Jenkinson, S Parkes, J M Birch, T O B Eden, P W James, P A McKinney, M S Pearce, R J Q McNally

**Affiliations:** 1 Institute of Health and Society, Newcastle University, Newcastle upon Tyne NE1 4LP, UK; 2 Paediatric Epidemiology Group, Centre for Epidemiology and Biostatistics, University of Leeds, Leeds LS2 9JT, UK; 3 West Midlands Regional Children's Tumour Registry, Birmingham Children's Hospital, Birmingham B4 6NH, UK; 4 Cancer Research UK Paediatric and Familial Cancer Research Group, University of Manchester, Manchester M27 4HA, UK; 5 Academic Unit of Paediatric and Adolescent Oncology, c/o Teenage Cancer Trust Young Oncology Unit, Christie Hospital, University of Manchester, Manchester M20 4BX, UK

**Keywords:** bone cancer, children, Ewing sarcoma, incidence, osteosarcoma, survival

## Abstract

There is a paucity of population-based studies examining incidence and survival trends in childhood bone tumours. We used high quality data from four population-based registries in England. Incidence patterns and trends were described using Poisson regression. Survival trends were analysed using Cox regression. There were 374 cases of childhood (ages 0–14 years) bone tumours (206 osteosarcomas, 144 Ewing sarcomas, 16 chondrosarcomas, 8 other bone tumours) registered in the period 1981–2002. Overall incidence (per million person years) rates were 2.63 (95% confidence interval (CI) 2.27–2.99) for osteosarcoma, 1.90 (1.58–2.21) for Ewing sarcoma and 0.21 (0.11–0.31) for chondrosarcoma. Incidence of Ewing sarcoma declined at an average rate of 3.1% (95% CI 0.6–5.6) per annum (*P*=0.04), which may be due to tumour reclassification, but there was no change in osteosarcoma incidence. Survival showed marked improvement over the 20 years (1981–2000) for Ewing sarcoma (hazard ratio (HR) per annum=0.95 95% CI 0.91–0.99; *P*=0.02). However, no improvement was seen for osteosarcoma patients (HR per annum=1.02 95% CI 0.98–1.05; *P*=0.35) over this time period. Reasons for failure to improve survival including potential delays in diagnosis, accrual to trials, adherence to therapy and lack of improvement in treatment strategies all need to be considered.

Malignant bone tumours are the seventh most common group of malignancies in children, comprising around 3–5% of cancers at ages 0–14 years (Parkin *et al*, 1998) with an age standardised incidence of approximately 5 per million person years in the United Kingdom (Stiller, 2007). The majority of these tumours are osteosarcoma or Ewing sarcoma (Stiller *et al*, 2006a), in the United Kingdom 54% being osteosarcoma, 39% Ewing sarcoma, 2% chondrosarcoma and 5% other types (Stiller, 2007). Worldwide, trends in incidence rates for childhood bone tumours have remained constant (McNally *et al*, 2001; Stiller *et al*, 2006a; Magnanti *et al*, 2008), although a few studies have reported increases (Ajiki *et al*, 1994; Gurney *et al*, 1996). The substantial improvement in survival over the past 40 years (Arndt *et al*, 2007) has been attributed to chemotherapy (Stiller *et al*, 2006a). Among European countries (19 countries), 5-year survival for all childhood bone tumours improved from 37% in 1978–1982 to 63% in 1993–1997. For osteosarcoma and Ewing sarcoma, 5-year survival also improved from 37 to 61% and from 34 to 66%, respectively over this period (Stiller *et al*, 2006a). We describe the incidence and survival from bone tumours at ages 0–14 years in northern England and the West Midlands during the period 1981–2002, updating previous analyses (Cotterill *et al*, 2000; Stiller *et al*, 2006c).

## Materials and methods

**Figure 1:**
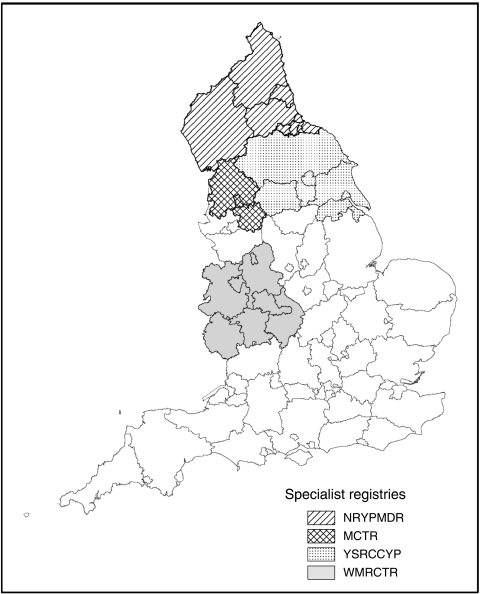
Geographical areas covered by the specialist registries.

Data were included for all children registered during the period 1981–2002 by the Northern Region Young Persons' Malignant Disease Registry (NRYPMDR), the Yorkshire Specialist Register of Cancer in Children and Young People (YSRCCYP), the West Midlands Regional Children's Tumour Registry (WMRCTR) and the Manchester Children's Tumour Registry (MCTR). These are specialist registries that record cases of cancer in children and young adults, and together cover around 35% of the 0- to 14-year-old population in England (2001 combined census population for 0–14 year olds approximately 3 250 000) (Figure 1). All four registries are population based and have very high levels of ascertainment due to rigorous data collection procedures. Methods of follow-up vary according to the individual registries. At the NRYPMDR, children are actively followed up through general practitioners and hospital records. At the YSRCCYP, children are actively followed up through general practitioners and hospital records every 2 years to determine health status, treatment and adverse outcomes such as second malignancies or relapse. Cross-checks with the national clinical trial databases are undertaken to ensure trial entry status is complete. At the WMRCTR, follow-up methods are both clinical and postal: surviving patients are seen annually or every 2 years in clinic, and those who do not attend have a postal questionnaire sent to their GP every 3 years. At the MCTR, for the first 35 years (1954–late 1980s) active follow-up was carried out by writing to hospitals, GPs and, in some instances, patients or their parents. The MCTR also received discharge summaries from the Christie Hospital and Royal Manchester Children's Hospital. From the 1980s onwards, active follow-up was ceased as cases were flagged at the National Health Service Central Register (NHSCR) and they continue to receive discharge summaries and follow-up clinic letters from the two main hospitals. The percentage of cases lost to follow-up are as follows: NRYPMDR 0.72%, YSRCCYP less than 0.5%, WMRCTR 4.2% (although tracing through the NHS Strategic Tracing Service has not revealed any deaths in this group), MCTR less than 10% (Birch, 1988; Muir *et al*, 1992; Cotterill *et al*, 2000; Feltbower *et al*, 2007).

Malignant bone tumours were defined as all those neoplasms in group VIII of the International Classification of Childhood Cancer (ICCC) (Steliarova-Foucher *et al*, 2005). Primary malignant bone tumours registered between 1981 and 2002 in patients aged 0–14 years at the time of diagnosis were extracted from the four participating English cancer registries. The diagnostic subgroups included osteosarcomas (ICCC subgroup VIIIa), chondrosarcomas (ICCC subgroup VIIIb), Ewing sarcomas (ICCC subgroup VIIIc), specified malignant tumours (ICCC subgroup VIIId) and unspecified malignant tumours (ICCC subgroup VIIIe). Secondary and benign bone tumours were excluded from this study. Information available for each case included demographic data (gender, date of birth), information on the tumour (diagnosis date, site and for NRYPMDR only, stage) and information on follow-up (current status, date of death, date of last follow-up).

### Statistical analysis

Age-specific incidence rates per million person years were calculated based on mid-year population estimates for the study region obtained from Office for National Statistics. Age-standardised incidence rates (ASR) were calculated based on the standard world population (Smith, 1992). Poisson regression models were used to assess the effects on incidence rate of time period for diagnosis (1981–1988, 1989–1995, 1996–2002), age group (0–4, 5–9, 10–14), gender and registry. Regression coefficients and corresponding 95% confidence intervals (CIs) are reported. Five-year survival rates were calculated for all cases diagnosed between 1981 and 2000, as follow-up was only available for all cases until the end of 2005. Survival rates were calculated using Kaplan–Meier estimation (Kaplan and Meier, 1958) and the differences in survival between diagnostic groups were assessed using log-rank tests. Cox Proportional Hazards regression analysis was used to model the probability of survival in relation to age, gender and year of diagnosis. Statistical significance was taken to be *P*<0.05 in all analyses. All statistical analyses were performed using Stata version 10.

## Results

There were 374 patients aged 0–14 years diagnosed with a malignant bone tumour in the study region between 1981 and 2002. The ASR over the study period was 4.84 per million for all 0–14 year olds. The number of cases and age-specific incidence rates by gender and disease type are shown in Table 1. There was no statistical evidence for any gender differences in total bone tumour incidence or within any of the bone tumour subgroups. The most common malignant bone tumours diagnosed were osteosarcoma (206 cases) and Ewing sarcoma (144 cases). These comprised 55.1 and 38.5% of diagnoses, respectively. Other subgroups were diagnosed in smaller numbers, with 16 chondrosarcoma cases (4.3% of diagnoses) and 8 other bone tumours (2.1%). Age-standardised incidence rates per million population years for each of the bone tumour subgroups were 2.63 (95% CI 2.27–2.99) for osteosarcoma, 0.21 (95% CI 0.11–0.31) for chondrosarcoma, 1.90 (95% CI 1.58–2.21) for Ewing sarcoma and 0.10 (95% CI 0.04–0.20) for the group comprising other specified and unspecified tumours.

Bone cancer incidence was not uniform across age groups (Table 1). Poisson regression models showed that after adjustment for gender and time period, incidence was highest at age 10–14 years. The incidence rate ratios (IRRs) for age group 10–14 years relative to 0–4 years were 13.21 (95% CI 6.48–26.94) for osteosarcoma; 2.88 (95% CI 1.05–7.93) for chondrosarcoma; 3.76 (95% CI 2.23–6.33) for Ewing sarcoma and 5.19 (95% CI 1.05–25.70) for other bone tumours. Incidence rates were also higher in the age group 5–9 years than 0–4 years for osteosarcoma and Ewing sarcoma. The IRRs for age group 5–9 years relative to 0–4 years were 5.96 (95% CI: 2.85–12.47) for osteosarcoma and 2.03 (95% CI: 1.16–3.55) for Ewing sarcoma.

Incidence did not differ between males and females for osteosarcoma (*P=*0.14), chondrosarcoma (*P*=0.37), Ewing sarcoma (*P*=0.98) or other bone tumours (*P*=0.94). Incidence rates were also similar in all four registries.

The results of the analyses of time trends in bone tumour incidence during 1981–2002 are shown in Table 2. After adjustment for age and gender, there was evidence that the incidence rate for Ewing sarcoma decreased on average by 3.1% (95% CI 0.6–5.6) each year (*P*=0.04). This rate of decline was not affected by gender (*P*=0.64). There was no evidence of any change over time for osteosarcoma (*P*=0.64), chondrosarcoma (*P*=0.72) or other bone tumours (*P*=0.79).

**Figure 2:**
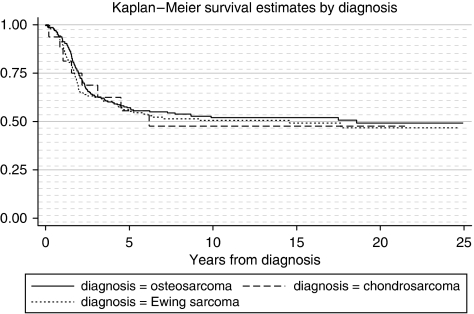
Kaplan–Meier survival by diagnosis group. Log-rank test for differences between diagnostic groups; *P*=0.85.

Five-year survival rates by period of diagnosis by bone tumour subgroup are shown in Table 3. In total, 57.8% (95% CI 52.5–63.0) of 0–14 year olds diagnosed in the study region during 1981–2000 survived for at least 5 years from the date of diagnosis. The 5-year survival rates for the bone tumour subgroups are respectively 58.8% (95% CI 51.8–65.9) for osteosarcoma, 53.3% (95% CI 26.6–78.8) for chondrosarcoma, 54.9% (95% CI 46.4–63.3) for Ewing sarcoma and 100% for other bone tumours. We found no difference in survival rate between diagnostic groups (log-rank test: *P*=0.85) (Figure 2).

**Figure 3:**
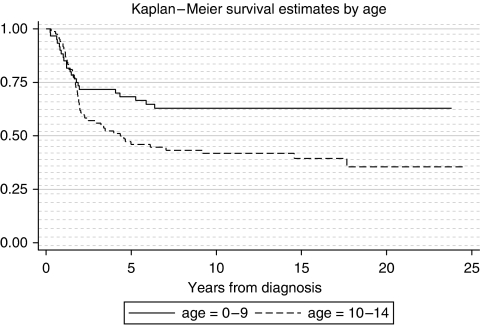
Kaplan–Meier survival for Ewing sarcoma by age. HR (comparing ages 10–14 years with ages 0–9 years)=1.86 (95% CI 1.12–3.07; *P*=0.02).

A Cox Proportional Hazards regression model for survival showed that for Ewing sarcoma the risk of death was higher for cases aged 10–14 years than for those aged 0–9 years (hazard ratio (HR)=1.86 95% CI 1.12–3.07; *P=*0.02) (Figure 3). After adjusting the model for age, the risk of death decreased by 5% for each year of the study period (HR=0.95 95% CI 0.91–0.99; *P*=0.02). Survival rates were similar for male and female Ewing sarcoma patients (*P=*0.79). The analysis of 5-year survival rates over the study period for Ewing sarcoma shows when improvement occurred (overall survival 37.5% of cases diagnosed in 1981–1987, 95% CI 24.8–50.1; 66.0% for 1988–1994, 95% CI 52.4–79.5; and 70.0% for cases diagnosed in 1995–2000, 95% CI 53.6–86.4) (Table 3).

For osteosarcoma, there was no evidence that gender (*P*=0.54), age (*P*=0.45) or year of diagnosis (*P*=0.35) had any effect on survival.

For chondrosarcoma, neither gender (*P*=0.23) nor age (*P=*0.16) had any effect on survival. There was marginally significant evidence that year of diagnosis had an effect on survival for chondrosarcoma, with risk of death decreasing by 10% for each year of the study period (HR=0.90, 95% CI: 0.80–1.02; *P=*0.09).

There was no evidence that disease site had any effect on survival when lower limbs were compared with all other sites (osteosarcoma *P=*0.52; chondrosarcoma *P*=0.71; Ewing sarcoma *P*=0.43). As stage was only available from the NRYPMDR, there were too few patients for any further analyses to be conducted on survival by stage.

Inspection of Schoenfeld residuals showed that all Cox regression models met the assumption of proportional hazards.

## Discussion

This study of childhood bone cancer in 0–14 year olds in northern England and the West Midlands during the period 1981–2002 found an improvement in survival for Ewing sarcoma, but not for osteosarcoma.

The overall incidence rate was 4.84 per million, similar to that reported worldwide in countries with predominantly white populations (Parkin *et al*, 1998; Stiller *et al*, 2006a). Bone tumours in this age group have been previously reported to occur in the United Kingdom at an incidence rate of 5 per million (Stiller, 2007). In addition, the increasing preponderance with age, the equal occurrence of male and female cases in the ages 0–14 years and the predominance of osteosarcoma and Ewing sarcoma found in this study are also typical patterns reported in earlier studies (Parkin *et al*, 1998; Stiller *et al*, 2006a).

Incidence rates were highest at ages 10–14 years. This is consistent with previous literature, as it has been widely reported that incidence of malignant bone tumours peak during the time of puberty (with the peak occurring later in boys than in girls), when children are undergoing a growth spurt and bones experience rapid growth (Grovas *et al*, 1997; Martin *et al*, 1997; Stiller *et al*, 2006a).

Whereas no significant temporal change was found in the incidence of childhood osteosarcoma, chondrosarcoma, other specified or other unspecified bone tumours over the study period, there was a decrease in the incidence of Ewing sarcoma. Several recently published studies have reported that bone tumour incidence remains stable over time in spite of an overall increase in cases of childhood cancer (McNally *et al*, 2001; Stiller *et al*, 2006a; Magnanti *et al*, 2008), with one specifically reporting stable incidence of Ewing sarcoma for the time period 1973–2004 (Esiashvili *et al*, 2008). Our observed decrease in the incidence of Ewing sarcoma remains unexplained. Although this decrease may be real, the role of artefact cannot be excluded. In three of the four registries (NRYPMDR, YSRCCYP, WMRCTR), there were increases in the incidence of soft-tissue Ewing sarcoma/pPNET during the study period. Some tumours previously classified as Ewing may now be classified as soft tissue sarcoma.

Overall, 57.8% of cases survived for at least 5 years, osteosarcoma (58.8%) and Ewing sarcoma (54.9%). This is slightly lower than a recent large Europe-wide study using data from the Automated Childhood Cancer Information System project, which reported the 5-year survival rate as 61% for malignant bone tumours in children aged 0–14 years in Europe during 1988–1997, with 5-year survival of 59% for osteosarcoma and 62% for Ewing sarcoma (Stiller *et al*, 2006a). Our results are also lower than those reported in the EUROCARE-3 study analysis, which reported 66% 5-year survival for osteosarcoma and 69% for Ewing sarcoma during the time period 1990–1994 (Gatta *et al*, 2003). However, our results are in line with data from an earlier EUROCARE study, which reported 5-year survival for malignant bone tumours in children aged 0–14 years in England and Wales to be 43 and 45% for osteosarcoma and Ewing sarcoma, respectively, for 1978–1989, and 55 and 57% for 1985–1989 (Stiller *et al*, 2001). Poorer survival rates may be due to failure to improve accrual into trials.

Our data show that survival from bone tumours in northern England and the West Midlands is lower than that seen in other European countries. Survival data comparing European regions show northern England and the West Midlands to have lower 5-year survival than the northern, southern and western regions of Europe, with these reporting 66, 65 and 66% 5-year survival, respectively. Only eastern Europe, which has a 41% 5-year survival rate, performs worse than northern England and the West Midlands (Stiller *et al*, 2006a). Variations between countries may reflect differing treatment strategies.

Five-year survival from malignant bone tumours was lower in children in the older age group of 10–14 years than those under the age of 10 years. Lower survival in older age groups has been reported in several studies. However, these tended to compare survival in adolescents aged 15–19 years with children aged 0–14 years (Stiller *et al*, 2006a, 2006b). No significant survival differences by age were observed in both a recent UK based study analysing data from the National Registry of Childhood Tumours (Stiller, 2007) and in a US study analysing SEER data (Ries *et al*, 1999). However, our data support another US study of Ewing sarcoma, which reports better survival below the age of 10 years than at ages 10–19 years (Esiashvili *et al*, 2008).

Gender was not significant in relation to survival for osteosarcoma or Ewing sarcoma, as reported for Ewing sarcoma in the United Kingdom (Stiller *et al*, 2006c). However, a recent study of Ewing sarcoma from the United States of America has reported a higher 5-year survival rate in females than males (Esiashvili *et al*, 2008). In addition, higher survival rates for osteosarcoma have been reported in females than males in a few studies. This difference between genders observed in some studies (Scranton *et al*, 1975; Homa *et al*, 1991) may be due to poor response to chemotherapy among males (Bielack *et al*, 2002).

Survival for Ewing sarcoma increased over the time period 1981–2002, whereas survival for osteosarcoma and the other subgroups remained unchanged. Studies reporting increased survival in both Ewing sarcoma and osteosarcoma over time (Miller *et al*, 1995; Dama *et al*, 2006; Stiller *et al*, 2006a) were concerned with earlier time periods. The most recent study of survival trends in Ewing sarcoma reported an improvement over a 30-year period up until 2004 (Esiashvili *et al*, 2008). The late 1970s and early 1980s saw the introduction of chemotherapy into the treatment of childhood bone tumours in addition to local control through surgery and radiotherapy. This gave rise to an increase in survival from the disease in this time period (Stiller *et al*, 2006a). Treatment for childhood bone tumours has not markedly changed since the 1980s, which may explain the subsequent hiatus in survival improvement. However, the lack of progress in achieving improved survival is of concern and requires further investigation and intervention measures. The possible contribution of delay in diagnosis to the lack of improved survival for osteosarcoma should be recognised, as it has been confirmed that long diagnosis delays in bone tumours are common. These long symptom durations are due to both patients and health professionals. Several factors may influence diagnosis delays, for example, delays may be greater for those living in rural compared with urban areas (Schnurr *et al*, 2008), while the site and biology of the tumour may also determine the extent of delay. Ewing sarcoma patients are more likely to have a longer symptom interval than osteosarcoma due to its greater propensity for growth in the axial skeleton. Early diagnosis and treatment are necessary for securing a favourable outcome (Bielack *et al*, 2002).

The efficacy of UK treatment regimens also needs investigation. Despite the increase in survival for Ewing sarcoma, the United Kingdom still lags behind Europe, and one contributing factor may be due to the differing treatment in the United Kingdom. This issue has been studied in a comparative survival analysis between the United Kingdom and Germany, where lower overall 5-year survival rates were observed in UK patients compared with those treated in Germany. Local therapy practice appeared to be the only explanatory factor associated with this difference in survival. For example, a higher proportion of German patients were treated with combined radiotherapy and surgery, reflecting a more aggressive philosophy of treatment than in the United Kingdom (Lewis, 2006). A similar change in UK practice may therefore result in increased 5-year survival, reducing the gap in survival with the rest of Europe.

This study highlights the worse survival from bone tumours overall than in other western European countries, and that, in spite of a significant increase in survival from Ewing sarcoma, there has been no such improvement since 1981 from osteosarcoma.

## Figures and Tables

**Table 1:** Number of cases and incidence rates per million per year by diagnostic group, gender and age- group, 1981–2002^a^

		**Age 0–4 years**		**Age 5–9 years**		**Age 10–14 years**		**Age 0–14 years**
**Diagnostic group**	**Gender**	***N***	**Rate (95% CI)**	***N***	**Rate (95% CI)**	***N***	**Rate (95% CI)**	**ASR (95% CI)**
All types	All persons	28	1.52 (0.96, 2.08)	108	4.66 (3.78, 5.53)	238	9.88 (8.63, 11.14)	4.84 (4.35, 5.34)
	Male	15	1.59 (0.78, 2.39)	52	4.38 (3.19, 5.56)	116	9.39 (7.68, 11.10)	4.63 (3.96, 5.31)
	Female	13	1.44 (0.66, 2.23)	56	4.95 (3.65, 6.25)	122	10.40 (8.56, 12.25)	5.06 (4.34, 5.79)
Osteosarcoma	All persons	8	0.43 (0.13, 0.73)	60	2.59 (1.93, 3.24)	138	5.73 (4.78, 6.69)	2.63 (2.27, 2.99)
	Male	4	0.42 (0.01, 0.84)	26	2.19 (1.35, 3.03)	65	5.26 (3.98, 6.54)	2.37 (1.89, 2.84)
	Female	4	0.44 (0.01, 0.88)	34	3.01 (2.00, 4.02)	73	6.22 (4.80, 7.65)	2.91 (2.37, 3.46)
Chondrosarcoma	All persons	2	0.11 (0.01, 0.39)	4	0.17 (0.05, 0.44)	10	0.42 (0.16, 0.67)	0.21 (0.11, 0.31)
	Male	2	0.21 (0.03, 0.77)	2	0.17 (0.02, 0.61)	6	0.49 (0.18, 1.06)	0.26 (0.10, 0.42)
	Female	0	0.00 (0.00, 0.00)	2	0.18 (0.02, 0.64)	4	0.34 (0.09, 0.87)	0.16 (0.06, 0.34)
Ewing sarcoma	All persons	17	0.92 (0.48, 1.36)	43	1.85 (1.30, 2.41)	84	3.49 (2.74, 4.23)	1.90 (1.58, 2.21)
	Male	9	0.95 (0.33, 1.58)	24	2.02 (1.21, 2.83)	41	3.32 (2.30, 4.34)	1.91 (1.47, 2.35)
	Female	8	0.89 (0.27, 1.51)	19	1.68 (0.92, 2.43)	43	3.67 (2.57, 4.76)	1.88 (1.44, 2.33)
Other^b^	All persons	1	0.05 (0.00, 0.30)	1	0.04 (0.00, 0.24)	6	0.25 (0.09, 0.54)	0.10 (0.04, 0.20)
	Male	0	0.00 (0.00, 0.00)	0	0.00 (0.00, 0.00)	4	0.32 (0.09, 0.83)	0.09 (0.03, 0.24)
	Female	1	0.11 (0.00, 0.62)	1	0.09 (0.00, 0.49)	2	0.17 (0.02, 0.62)	0.11 (0.03, 0.29)

ASR=age-standardised incidence rates; CI=confidence interval.

a Rates data include 95% CI in parentheses.

b Other specified and unspecified tumours.

**Table 2:** Number of cases and ASR's per million per year by diagnostic group and time periods, 1981–2002^a^

		**1981–1988**		**1989–1995**		**1996–2002**	
**Diagnostic group**	**Gender**	***N***	**Rate (95% CI)**	***N***	**Rate (95% CI)**	***N***	**Rate (95% CI)**
All types	All persons	149	5.29 (4.43, 6.15)	126	5.41 (4.46, 6.35)	99	3.82 (3.07, 4.58)
	Male	66	4.58 (3.46, 5.70)	67	5.61 (4.26, 6.95)	50	3.80 (2.74, 4.86)
	Female	83	6.05 (4.73, 7.36)	59	5.20 (3.87, 6.53)	49	3.85 (2.77, 4.93)
							
Osteosarcoma	All persons	75	2.59 (2.00, 3.18)	73	3.11 (2.39, 3.82)	58	2.24 (1.66, 2.82)
	Male	33	2.21 (1.45, 2.97)	33	2.73 (1.80, 3.67)	29	2.20 (1.39, 3.00)
	Female	42	2.98 (2.07, 3.89)	40	3.50 (2.42, 4.59)	29	2.28 (1.45, 3.11)
							
Chondrosarcoma	All persons	7	0.28 (0.11, 0.58)	3	0.13 (0.03, 0.37)	6	0.22 (0.08, 0.48)
	Male	3	0.26 (0.05, 0.77)	3	0.25 (0.05, 0.72)	4	0.29 (0.08, 0.73)
	Female	4	0.30 (0.08, 0.77)	0	0.00 (0.00, 0.00)	2	0.15 (0.02, 0.54)
							
Ewing sarcoma	All persons	63	2.27 (1.71, 2.84)	49	2.13 (1.53, 2.73)	32	1.26 (0.82, 1.69)
	Male	28	1.98 (1.24, 2.72)	30	2.55 (1.63, 3.46)	16	1.24 (0.63, 1.86)
	Female	35	2.58 (1.72, 3.45)	19	1.70 (0.93, 2.46)	16	1.27 (0.64, 1.89)
							
Other^b^	All persons	4	0.15 (0.04, 0.39)	1	0.04 (0.00, 0.23)	3	0.11 (0.02, 0.32)
	Male	2	0.12 (0.01, 0.45)	1	0.08 (0.00, 0.45)	1	0.07 (0.00, 0.40)
	Female	2	0.18 (0.02, 0.65)	0	0.00 (0.00, 0.00)	2	0.15 (0.02, 0.54)

ASR=age-standardised incidence rates; CI=confidence interval.

a Rates data include 95% CI in parentheses.

b Other specified and unspecified tumours.

**Table 3:** Percentage 5-year survival by time period, diagnostic group and age^a^

		**1981–1987**	**1988–1994**	**1995–2000**
**Diagnostic group**	**Gender**	**Rate (95% CI)**	**Rate (95% CI)**	**Rate (95% CI)**
All types	All persons	51.13 (42.63, 59.62)	61.74 (52.86, 70.62)	62.37 (52.52, 72.21)
	Male	57.14 (44.18, 70.10)	59.70 (47.96, 71.45)	61.90 (47.22, 76.59)
	Female	46.75 (35.61, 57.90)	64.58 (51.05, 78.11)	62.75 (49.48, 76.01)
				
Osteosarcoma	All persons	60.61 (48.82, 72.39)	58.46 (46.48, 70.44)	57.14 (44.18, 70.10)
	Male	68.97 (52.13, 85.80)	58.06 (40.69, 75.44)	54.17 (34.23, 74.10)
	Female	54.05 (38.00, 70.11)	58.82 (42.28, 75.37)	59.38 (42.36, 76.39)
				
Chondrosarcoma	All persons	42.86 (9.90, 81.59)	50.00 (1.26, 98.74)	67.00 (22.28, 95.67)
	Male	66.67 (9.43, 99.16)	50.00 (1.26, 98.74)	75.00 (19.41, 99.16)
	Female	25.00 (0.63, 80.59)	0.00 (—)	50.00 (1.26, 98.74)
				
Ewing sarcoma	All persons	37.50 (24.82, 50.18)	65.96 (52.41, 79.50)	70.00 (53.60, 86.40)
	Male	36.36 (16.26, 56.47)	60.61 (43.93, 77.28)	71.43 (47.76, 95.09)
	Female	38.24 (21.90, 54.57)	78.57 (49.20, 95.34)	68.75 (46.04, 91.46)
				
Other^b^	All persons	100.00 (39.76, 100.00)	100.00 (2.50, 100.00)	100.00 (2.50, 100.00)
	Male	100.00 (15.81, 100.00)	100.00 (2.50, 100.00)	0.00 (—)
	Female	100.00 (15.81, 100.00)	0.00 (—)	100.00 (2.50, 100.00)

CI=confidence interval.

a Rates data include 95% CI in parentheses.

b Other specified and unspecified tumours.
